# Preoperative quality of life of patients with cleft lip and palate in Nigeria: a multicentre cross-sectional pilot study

**DOI:** 10.11604/pamj.2024.48.50.42111

**Published:** 2024-06-07

**Authors:** Afieharo Igbibia Michael, Adeola Adenike Olusanya, Chinedu Michael Okoli, Bardi Martins, Akintunde Joseph Akintayo, Ijeoma Onwuagha

**Affiliations:** 1 Department of Plastic, Reconstructive and Aesthetic Surgery, University College Hospital, Ibadan, Oyo State, Nigeria,; 2 Department of Oral and Maxillofacial Surgery, Afidea Medical Centre, Ibadan, Oyo State, Nigeria,; 3 Department of Burns and Plastic Surgery, National Orthopaedic Hospital, Enugu, Enugu State, Nigeria,; 4 Department of Oral and Maxillofacial Surgery, Amino Kano Teaching Hospital, Kano State, Nigeria,; 5 Department of Surgery, Jos University Teaching Hospital, Jos, Plateau State, Nigeria,; 6 Department of Surgery, University of Port Harcourt Teaching Hospital, Port Harcourt, Rivers State, Nigeria

**Keywords:** Cleft lip and palate, quality of life, patient-reported outcome

## Abstract

**Introduction:**

the objective of this study was to determine the quality of life (QoL) of the patient with a cleft lip or palate scheduled for surgery.

**Methods:**

this analytic multicenter cross-sectional study involved six participating Smile Train Partner Hospitals from five geopolitical zones of the country and three major ethnic groups. Patients with cleft lip or cleft palate aged between 8 to 29 years scheduled for repair were recruited. The main outcome measure was quality of life scores as measured by cleft Q.

**Results:**

thirty-four (females 18, males 16) patients were scheduled for surgery of either cleft lip n=7 (20.6%) or cleft palate n=27 (79.4). Patients scheduled for primary surgeries were more than those for secondary surgeries, 23 (68.7%) vs 10 (30.3%). Of the QoL scales, the speech distress score was the least (56.0 ± 22.6) and the psychological score highest (73.9 ± 15.8). All QoL mean scores except the psychological score fell below normative cleft Q scores. The psychological scores in males (80.9 ± 16.2) were significantly higher than in females (67.7 ± 12.9, p=0.01). Patients for lip repair had lower psychological scores than those for palatal repair (median=59 vs 73, p=0.01). Patients for palate repair demonstrated significantly lower speech function and distress scores than those for lip repair (p=0.01, p<0.01 respectively).

**Conclusion:**

most of the QoL measures in patients with cleft lip and palate in this study fell below normative values. Gender and cleft type affect the quality of life. A larger study is recommended to establish national normative data.

## Introduction

Globally, a patient with a cleft of the lip and/or the palate has functional and psychosocial limitations that are associated with the cleft type of specific deformity [1-3]. These limitations include problems with appearance, feeding, drinking, increased occurrence of chest and middle ear infections as well as speech deficits. Psychosocial limitations become apparent as the child becomes self-aware and could persist into adulthood if the defect is not repaired [1-4]. From these, the quality of life of the patient with a cleft can be significantly affected. Patient-reported outcomes, allow self-reporting of the impact of the deformity on the patient as well as the impact of the intervention.

The cleft Q is a cleft-specific patient-reported outcome measure that has been accepted by the International Consortium for health outcome measures as an objective patient-reported outcome measure [5]. It has been shown to be discriminatory enough between cleft types, and age groups and between patients who have and have not had cleft surgery [6]. The cleft Q was designed for patients with clefts from 8 to 29 years. It consists of three main domains: appearance, facial function, and health-related quality of life. These domains have subscales that ask about the typical challenges experienced by a patient with a cleft. Each subscale can be used independently and normative cleft Q scores for each scale and tailored to the cleft type, age, and gender of the patient have been established [5].

The ability of the patient to provide a self-reported outcome gives the cleft care provider a basis for the determination of an appropriate intervention. Utilization of self-reported outcomes also enables healthcare providers to judge the effectiveness of any intervention administered [7,8].

The absence of a unified quality-of-life outcome tool makes it difficult for cleft surgeons to consistently review outcomes within their institutions and compare outcomes across institutions globally. This is particularly the situation in low- and middle-income countries where outcome measures are largely institutionalized. Therefore, this multicenter pilot study seeks to determine the preoperative quality of life of the patient with a cleft across different regions in a low-resource setting using a standardized instrument, the cleft Q.

## Methods

**Study design/setting:** this was an analytic cross-sectional study between January to August 2022. Ethical approval was obtained from the ethical review boards of the participating institutions. Patients were recruited from six Smile Train Partner Hospitals in five of the six geopolitical zones of the country and representing the three main cultures in Nigeria. Smile Train provides free surgeries for all patients with clefts in Nigeria who are treated at partner hospitals.

**Participants:** the inclusion criteria were patients with cleft lip with or without cleft palate and cleft palate aged between 8 to 29 years who were to receive surgery for repair of the cleft lip or palate. Patients with a cleft lip who were below eight years or above 29 years of age were excluded from the study. Patients with syndromic clefts were also excluded. The patients were recruited consecutively from the cleft clinics of the participating institutions. Written informed consent was obtained from all participants above 18 years and from parents of participants less than 18 years.

**Data:** the study instruments were a study questionnaire to obtain the sociodemographic and clinical characteristics of the patients and the cleft Q. The study instruments were self-administered, or researcher-administered for non-English speaking patients. The participants filled out the cleft Q scales which corresponded to their cleft-related surgery. All participants filled the quality-of-life subscales. A licensed agreement to use the cleft Q was obtained from McMaster University. All study sites either collected hardcopy data (study questionnaire and cleft Q) which were transferred to a Research Electronic Data Capture (RED Cap) mobile application on study tablets or entered data directly onto the offline mobile application. These data were subsequently uploaded to the web-based application of the host institution.

**Outcome:** the primary outcome measure was the QoL scores. These were the Rash transformed cleft Q sub-scale (psychological, school life, social life, speech distress) scores from the health-related quality of life domain.

**Bias:** we circumvented research bias amongst the children by ensuring that the questionnaire was administered by the research assistants without any interference from the parents. Also, any participant who did not fully understand the questions was allowed to seek clarification from the research assistant.

**Study size:** we determined our full-scale size to be 218 using the Cochran formula. For a pilot study, 10-20% of the full-scale size is recommended. Additionally, a sample size of at least 30 is recommended as appropriate for feasibility/pilot studies [9]. We used a sample size of 34.

**Quantitative variables:** the scores for each response on a specific scale were summed up to give a raw score. These raw scores were then transformed to a score from 0-100 using the conversion table provided by the authors. The derived QoL subscale scores were compared with established normative values.

**Data analysis:** data was exported from RED Cap and analyzed using Stata version 17.0 (Stata Corp, Texas, USA) statistical software. Categorical sociodemographic, and clinical variables were presented as frequencies and percentages, and continuous variables were presented as mean ± standard deviation. The normality of continuous variables was tested with the Shapiro-Wilk test. Relationships between independent variables and primary outcome measures were analyzed using Student´s t-test, Mann-Whitney U test, and one-way analysis of variance. A P-value < 0.05 was set as statistically significant.

## Results

**Participants:** thirty-four patients were recruited. All patients completed the questionnaires. There were no missing variables.

### Descriptive data

**Sociodemographic and clinical characteristics of participants:** there was a slightly higher female preponderance, 18 (52.9%). Patients from the three major ethnic groups in the country were represented. Most of the patients were Christians, 22 (64.7%). Other sociodemographic characteristics are depicted in Table 1. Most of the patients were scheduled to have repair of the cleft palate, N=27 (79.4%). Most of these were primary repairs, 23 (69.7%). More patients had bilateral clefts, 17 (51.5%). The clinical characteristics of the patients are shown in Table 2.

**Table 1:** sociodemographic characteristics of preoperative patients with clefts (8-29 years) recruited from six hospitals in Nigeria from January to August 2022

Variables	Frequency (%)
Age of patient (years), median (range)	14.5 (8-29)
**Gender**	
Male	16 (47.1)
Female	18 (52.9)
**Age group**	
< 10 years	9 (26.5)
10-19 years	14 (41.2)
≥20 years	11 (32.4)
**Ethnicity**	
Igbo	9 (26.5)
Hausa	10 (29.4)
Yoruba	8 (23.5)
Others	7 (20.6)
**Religion**	
Christianity	22 (64.7)
Muslim	12 (35.3)
**Education of patient**	
No formal	1 (3.0)
Primary	12 (36.4)
Secondary	12 (36.4)
Tertiary	8 (24.2)
**School type**	
Public	18 (56.3)
Private	14 (43.7)
**Fathers highest education**	
No formal	7 (20.6)
Primary	4 (11.8)
Secondary	10 (29.4)
Tertiary	13 (38.2)
**Mothers highest education**	
No formal	6 (17.7)
Primary	8 (23.5)
Secondary	11 (32.4)
Tertiary	9 (26.5)
**Family setting**	
Monogamous	28 (82.4)
Polygamous	6 (17.6)
**House ownership**	
Self-owned	19 (55.9)
Rented	15 (44.1)

**Table 2:** clinical details of preoperative patients with clefts (8-29 years) recruited from six hospitals in Nigeria from January to August 2022

Variable	Frequency (%)
**Type of surgery planned**	
Cleft lip repair	7 (20.6)
Cleft palate repair	27 (79.4)
Primary	23 (69.7)
Secondary	10 (30.3)
**Side of cleft**	
Right	3(9.1)
Left	4(12.1)
Bilateral	17(51.5)
Not applicable	9 (27.3)
**Previous cleft-related surgery**	
Yes	17 (51.5)
No	16 (48.5)
**Quality of life scores (mean ± SD)**	
Psychological function	73.9 (15.8)
School function	64.7 (14.3)
Social function	67.2 (16.7)
Speech distress	56.0 (22.6)
**Facial function scores, mean (SD)**	
Speech function	46.4 (28.2)
Eating and drinking, median (range)	31.5 (11-36)

**Outcome data:** the psychological function had the highest mean score 73.9 (±15.8) while the speech distress had the lowest mean score 56.0 (±22.6). The outcome scores are shown in Table 2.

### Main results

**Relationship between sociodemographic and clinical variables and primary outcomes:** although participants who were younger than 10 years or older than 20 years had lower scores in the speech distress and speech function subscales than participants who were between 10 and 20 years, this was not statistically significant (Table 3). Male participants had statistically significantly (p=0.01) higher psychological function scores than female participants despite having lower scores in speech function and speech distress as compared to the females. The Igbo ethnic group had the highest scores in all the quality-of-life subscales. None of them however reached statistical significance. Lower speech function scores were seen among the Yoruba ethnic group. Religion bore no relationship with the quality-of-life scores. Participants who attended public schools had higher scores in school function as compared to those who attended private schools, but this was not statistically significant (p=0.07). Neither family setting nor home ownership had any relationship with the quality-of-life scores.

**Table 3:** the association between sociodemographic/clinical characteristics and quality-of-life scores of preoperative patients with clefts (8-29 years) recruited from six hospitals in Nigeria from January to August 2022

Characteristics	Psychological function	p-value	School function	p-value	Social function	p-value	Speech distress	p-value	Speech function	p-value
Age	-0.2324	0.19	0.2124	0.22	-0.1599	0.37	-0.1826	0.30	0.1093	0.54
**Gender**										
Male	80.9 (16.1)	0.01	67.1 (15.7)	0.58	70.3 (18.3)	0.32	50.4 (35.6)	0.18	39.6 (23.1)	0.19
Female	67.7 (12.9)		64.4 (13.2)		64.5 (15.1)		60.9 (20.7)		52.3 (31.5)	
Religion										
Christian	74.0 (16.6)	0.9	64.4 (17.0)	0.4	69.1 (18.1)	0.37	55.7 (25.1)	0.93	48.9 (28.7)	0.48
Muslim	73.6 (14.9)		68.0 (7.0)		63.7 (13.9)		56.4 (18.3)		41.7 (24.0)	
**School type**										
Public	70.2 (14.1)	0.17	69.3 (14.1)	0.07	67.8 (16.8)	0.97	58.7 (22.7)	0.7	47.4 (28.0)	0.96
Private	78.2 (17.9)		60.4 (13.2)		67.6 (17.9)		55.8 (22.3)		47.9 (30.4)	
**Family setting**										
Monogamous	78.0 (16.3)	0.37	65.1 (15.7)	0.60	68.9(18.0)	0.21	54.3 (23.7)	0.35	45.2 (27.4)	0.6
Polygamous	68.5 (13.5)		68.5 (2.5)		59.3 (3.5)		64.0 (16.1)		51.8 (33.8)	
**House ownership**										
Self-owned	72.4 (15.5)	0.54	67.1 (12.0)		64.8 (14.5)	0.35	55.5 (18.9)	0.90	45.8 (22.1)	0.9
Rented	75.8 (16.5)		63.9 (17.0)		70.3 (19.3)		56.5 (27.3)		47.0 (35.3)	
**Scheduled Surgery**										
Cleft lip repair	64.0 (16.6)	0.06	68.9 (18.0)		67.4 (22.4)	0.97	78.7 (26.2)	0.002	78.9 (31.4)	0.000
Cleft palate repair	76.4 (14.9)		64.9 (13.4)		67.1 (15.4)		50.1 (17.8)		37.9 (20.6)	
Primary	75.3 (16.5)	0.6	69.1 (14.5)	0.06	70.0 (17.0)	0.25	55.1 (24.1)	0.85	46.0 (29.1)	0.70
Secondary	72.1 (14.8)		58.9 (11.6)		62.6 (15.6)		53.5 (15.4)		41.9 (22.5)	
**Previous cleft-related surgery**										
Yes	73.8 (14.6)	0.83	65.6 (15.3)	0.86	66.0 (16.1)	0.55	54.5 (20.1)	0.98	41.6 (21.1)	0.50
No	74.9 (17.6)		66.5 (13.8)		69.9 (17.6)		54.8 (23.7)		48.1 (32.5)	

Participants with cleft lip had lower psychological function scores than those with cleft palate (p=0.06). The speech function and speech distress scores of participants with cleft palate were significantly lower than participants with cleft lip, p<0.001 and P=0.002 respectively.

**Figure 1:**
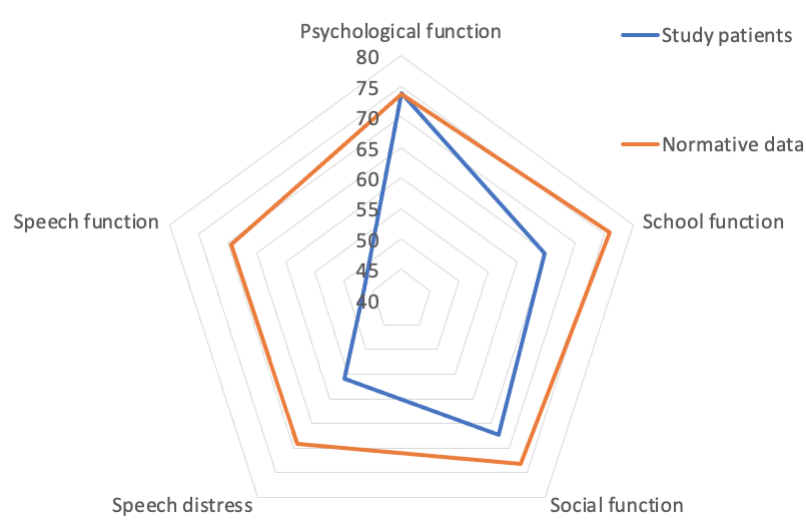
comparison of mean quality of life domain scores obtained from preoperative patients with clefts (8-29 years) recruited from six hospitals in Nigeria from January to August 2022 with normative data obtained from the cleft Q

**Figure 2:**
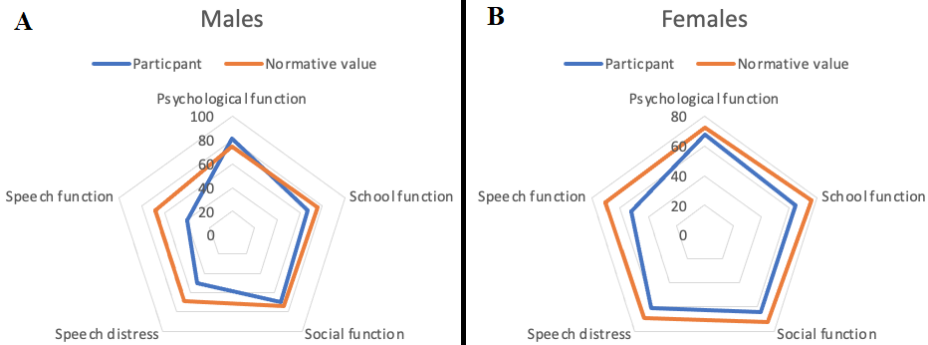
comparison of mean quality of life scores in male (A) and female (B) preoperative patients with clefts (8-29 years) recruited from six hospitals in Nigeria from January to August 2022 with normative data obtained from the cleft Q

**Figure 3:**
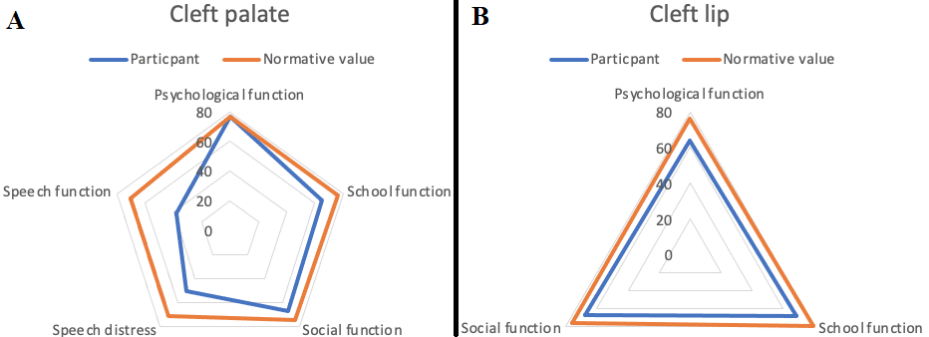
cleft type (cleft palate (A) and cleft lip (B)) matched comparison of preoperative patients with clefts (8-29 years) recruited from six hospitals in Nigeria from January to August 2022 with normative data obtained from the cleft Q

**Comparison between participant´s quality-of-life scores and normative values:** the overall matched comparisons for the quality-of-life scores showed only the psychological subscale score matched the normative value. All other scores fell below the normative values. These scores were lowest for speech function (Figure 1). When matched for gender, the male participants in this study had higher psychological scores than normative scores but lower scores for other subscales (Figure 2A). Female participants in this study had lower quality-of-life scores in this study when compared to the scores for females in the normative values. The cleft type matched comparisons with the normative values and demonstrated a comparable psychological subscale score in patients who had cleft palate (Figure 2B). All other quality-of-life subscale scores in patients with either cleft palate or cleft lip fell below the normative values (Figure 3 (A,B)). This was however less in patients with cleft lip (Figure 3B).

## Discussion

We set out to determine the quality of life of patients scheduled for either cleft lip or cleft palate repair and compare these values with established normative values. We have demonstrated that gender and cleft type affect the quality of life. Ethnicity also tended to impact the quality of life of these patients. Only the psychological function score of patients (males) in this study approximated that of the normative scores. All other quality-of-life scores fell below the normative values. This study has buttressed the importance of patient-reported outcome measures and revealed the directions a full-scale study should take. Being a pilot study, the results cannot be generalizable which is a limitation of the study.

Previous studies have reported that sociodemographic and economic factors affect the health-related quality of life of the patient with a cleft [1,10-13]. Herkrath *et al*. identified low socioeconomic status, being a female, and type of cleft as determinants of poor health-related quality of life in patients with cleft lip and palate [13]. Just as was demonstrated in the psychometric and production of normative values for the cleft Q [5], we also found that patients with a visible cleft (cleft lip) had lower psychological scores. In addition, females had lower scores than males. This lower rating by females was also recorded in the study by Sinko *et al*. [2]. Males probably have more adaptive mechanisms to disability than females. A study from a Chinese population however found no influence of gender on the psychological profile of their patients with cleft lip and palate [14]. Similarly in a Dutch-speaking population, no gender influences of the cleft were seen on their psychosocial wellbeing [15]. Psychosocial interventions for cleft care may therefore need to be targeted more at females with cleft.

Similar to our study, Ahmed *et al*. [4] identified an association between the type of orofacial cleft and psychological function as we did in this study. Having a visible deformity opens the patient to stigmatization [10]. Therefore, these patients with the cleft type lower score would benefit more from psychological intervention programs in addition to the management of the deformity. Ahmed *et al*. [4] found no association between quality-of-life scores and the educational level of the patient. Their study population was like ours with a mean age of 12 years as compared to our median age of 14 years. We also found no relationship between the educational status of the patients and their quality-of-life scores. We did notice however that the school function scores in public schools were higher than in patients who attended private schools. How contributory stigmatization is to these findings remains to be investigated.

The cleft Q has been reported to be able to demonstrate an improvement in the quality of life after surgery. Marcusson *et al*. reported that patients with a repaired cleft of the lip and palate were able to perform activities of daily living adequately when compared to controls without deformity [16]. These patients in their study however still reported impairments in social life and wellbeing. It is expected that the patients in our study who have been scheduled for surgery should demonstrate improvement from the preoperative scores. However, from previous reports psychosocial maladjustments may still persist even after cleft repair. This buttresses the need for psychosocial support during the management of patients with cleft [12].

Previous studies have shown a significant relationship between ethnicity and quality of life in patients with cleft [17,18]. A study in New Zealand reported poorer quality of life scores in children of Pacific Island [18] descent while Broder *et al*. reported a significant relationship between ethnicity and the quality of life in patients with cleft from their study in the United States of America [17]. Our study showed that the Igbo ethnic group had consistently higher scores than other ethnic groups. The patients in this study had high psychological scores despite having low scores in other domains. While we are unable with this study to determine the reason for this it suggests that there could be other social factors that may be contributory to this and should be explored.

The cleft Q can be used to report on the quality of life of the patient with a cleft in low-resource settings and it was able to discriminate between cleft types in this study. Patients scheduled for palatal repair had significantly higher speech distress and speech function scores than patients scheduled for lip repair. This clearly demonstrates alignment between the pathology of the deformity and the scores.

The ability to be able to measure outcomes that matter to the patient is integral to providing quality care. Quality of life measures have become a core component of clinical care outcomes, inclusive of cleft care [7,8,19-21]. The importance of comprehensive cleft care on a patient with a cleft is centered around ensuring this care is individualized, producing individuals who are well-integrated into society despite their deformity. Every cleft care provider is now encouraged to think beyond a specialty-centered treatment to the holistic care of the patient with a cleft.

**Limitation:** as a pilot study quality of life score obtained in these patients cannot be generalized.

## Conclusion

This pilot study had demonstrated low quality of life sores in patients with unrepaired clefts of the lip and palate. These scores fall below the normative cleft Q scores. Cleft type, gender, and possibly ethnicity are factors that affect the quality of life and should be targeted in interventions. The utilization of the same instrument across different geographic zones in the country shows potential for its use as a unified standardized instrument. This will foster further collaborative work. A larger cleft-specific population study across the country will further establish normative data for the country against which cleft care interventions can be compared.

### What is known about this topic

- *Having a cleft affects the quality of life of the individual;*
- *Patient-reported outcome measures are integral to providing optimal care*.

### What this study adds

- *Cleft-related quality of life outcome scores in a homogenous African population;*
- *Female gender have poorer quality of life scores than males so females should be a focus of psychosocial interventions*.
